# Effects of Internet-Based Guided Self-Help Problem-Solving Therapy for Adolescents with Depression and Anxiety: A Randomized Controlled Trial

**DOI:** 10.1371/journal.pone.0043485

**Published:** 2012-08-31

**Authors:** Willemijn Hoek, Josien Schuurmans, Hans M. Koot, Pim Cuijpers

**Affiliations:** 1 Department of Clinical Psychology, VU University, Amsterdam, The Netherlands; 2 Developmental Psychology, VU University, Amsterdam, The Netherlands; 3 EMGO+ Institute, Amsterdam, The Netherlands; The University of Queensland, Australia

## Abstract

**Background:**

Symptoms of depression and anxiety are highly prevalent in adolescence and they are the cause of considerable suffering. Even so, adolescents are not inclined to seek professional help for emotional problems. Internet-based preventive interventions have been suggested as a feasible method of providing appropriate care to adolescents with internalizing symptoms. The objective of this study was to evaluate the effects of preventive Internet-based (guided) self-help problem-solving therapy (PST) for adolescents reporting mild to moderate symptoms of depression and/or anxiety as compared to a waiting list control group (WL).

**Methodology/Principal Findings:**

A total of 45 participants were randomized to the 2 conditions. PST consisted of 5 weekly lessons. Participants were supported by e-mail. Self-report measures of depression and anxiety were filled in at baseline and after 3 weeks, 5 weeks, and 4 months. Of the 45 participants, 28 (62.2%) completed questionnaires after 3 weeks, 28 (62.2%) after 5 weeks, and 27 (60%) after 4 months. Hierarchical linear modeling analyses revealed overall improvement over time for both groups on depressive and anxiety symptoms. However, no significant group x time interactions were found. No differences were found between completers and non-completers.

**Conclusions/Significance:**

Results show that depressive and anxiety symptoms declined in both groups. No support was found, however, for the assumption that Internet-based PST was efficacious in reducing depression and anxiety in comparison to the waiting list control group. This finding could represent lack of power.

**Trial Registration:**

Netherlands Trial Register NTR1322

## Introduction

Despite the high prevalence and impact of depression and anxiety in adolescence [1], [2], many young people do not seek professional help for emotional problems [3]. Internet-based interventions, however, may enable treatment consideration and participation. Adolescents are particularly comfortable interacting within the computer environment [4], and Internet-based interventions may reduce stigma, maximize autonomy, save travelling time, provide low cost access, reduce waiting-lists, abolish the need to schedule appointments with a therapist, and reduce objections like lack of willingness to talk to a stranger about personal problems [5], [6].

Studies on adolescent Internet-based interventions for depression or anxiety are scarce (see [4], [7], [8]) and the effectiveness of merely two programs was examined in randomized controlled trials (RCTs) [9], [10], [11]. These concern a five session cognitive behavior therapy (CBT) based intervention program (MoodGYM) in preventing and reducing the symptoms of depression and anxiety in an adolescent school-based population, and a six session guided CBT group course (Master Your Mood) for adolescents aged 16 to 25 years with depressive symptoms. Previous controlled pilot trials of the MoodGYM intervention in two private single-sex schools demonstrated benefits on self-reported depression for adolescent boys who completed more modules [9] and for girls at follow-up [10]. A previous study with a more diverse sample reported decreases in anxiety symptoms for the total sample and decreases in depression for males only [7]. The online group course Master Your Mood was effective in reducing depressive and anxiety symptoms and in increasing mastery in young people [11].

As far as we know, there is no study which evaluates Internet-based problem-solving therapy (PST) for adolescents. PST is aimed at determining the major goals in life, investing energy only in those problems that are related to what matters and learning to accept those situations that cannot be changed, with problem-solving skills as the core element of this approach [12], [13]. Face-to-face problem-solving therapy has been found to be effective in a variety of problem areas [12], [14], and given high comorbidity and shared risk factors of depression and anxiety [15], the provision of an intervention that can concurrently decrease depressive and anxiety symptoms in adolescents would be appealing. A PST-based intervention that could be applied through the Internet was developed and studied in an adult population [13]. The intervention was successful in reducing adults' symptoms of depression, anxiety, and work related stress, and was consequently adapted for adolescents. The aim of the current study is to evaluate the effects of preventive Internet-based PST for adolescents reporting mild to moderate symptoms of depression and/or anxiety as compared to a waiting list control group.

## Methods

The protocol for this trial and supporting CONSORT checklist are available as supporting information; see Checklist S1 and Protocol S1.

### Participants

Participants were recruited through press releases, banners and advertisements on the Internet, advertisements in magazines, referral by school-doctors, through brochures and posters in schools, and through information to parents who are treated in mental health care institutions for depression and anxiety. Recruitment took place from January 2009 to May 2011. After application through a website, applicants received a brochure about this study and an informed consent form by email. If adolescents were younger than 18 years of age, parents received a brochure and an informed consent form by regular mail after application by the adolescent. Adolescents aged 17 years or younger needed parental consent for study participation. After receiving informed consent, adolescents received the baseline questionnaire by email.

To be included, applicants had to report mild to moderate depressive and/or anxiety symptoms (there was no lower limit for entering the study) and have sufficient knowledge of the Dutch language, access to the Internet, and an email address. At first, adolescents aged 12 to 18 years with depressive and/or anxiety symptoms were found eligible for this study. It was difficult to include adolescents in the study. Inasmuch as we had strong indications that parental consent requirements often withheld adolescents from participating [16] and because older adolescents showed interest in our intervention, the upper age limit was altered during the study and adolescents aged 19 to 21 years were also included from April 2010. Adolescents were excluded from the study if they were younger than 18 years of age and had no parental permission, were receiving other psychological treatment for mental health problems, had severe depressive symptoms (defined as a score above 40 on the Centre for Epidemiologic Studies Depression scale; CES-D [17]), had severe anxiety symptoms (indicated by a score above 14 on the anxiety subscale of the Hospital Anxiety and Depression Scale; HADS-A [18]), and/or had prominent suicide ideation (indicated by a score above 1 on the suicide item of the Beck Depression Inventory-II; BDI-II [19]). Respondents who were excluded due to the presence of severe symptoms received a telephone call in which they were advised to consult their general practitioner. If younger than 18 years, their parents were also informed by telephone. The study was approved by the Medical Ethics Committee of the VU University Medical Center, Amsterdam, the Netherlands. For a detailed description of the study and intervention we refer to the study protocol [20].

### Intervention

The PST intervention was web-based and participants were provided with a username and password to access the website. Every week an automated email was sent to participants to explain the contents and exercises for the coming week. Mental health care professionals and the authors (WH, JS) offered feedback on the completed exercises. This feedback was not therapeutic but was directed at mastering the proposed problem-solving strategies. PST consisted of three steps. First, respondents described what really mattered to them. Second, they wrote down their current worries and problems, and they divided these problems into three categories: (a) unimportant problems, (b) solvable problems, (c) problems which cannot be solved. For each of these three types of problems a different strategy was proposed to solve the problems or to learn to cope with the unimportant and unsolvable ones. This step is the most important element of PST. For the solvable problems, we proposed the following six-step procedure: (1) write a clear definition of the problem, (2) generate multiple solutions to the problem, (3) select the best solution, (4) work out a systematic plan for this solution, (5) carry out the solution, and (6) evaluate as to whether the solution has resolved the problem. During the third and last step, the respondents made a plan for the future in which they described how they would try to accomplish those things that matter most to them. The course took 5 weeks and consisted of one lesson a week.

### Design

**Figure 1:**
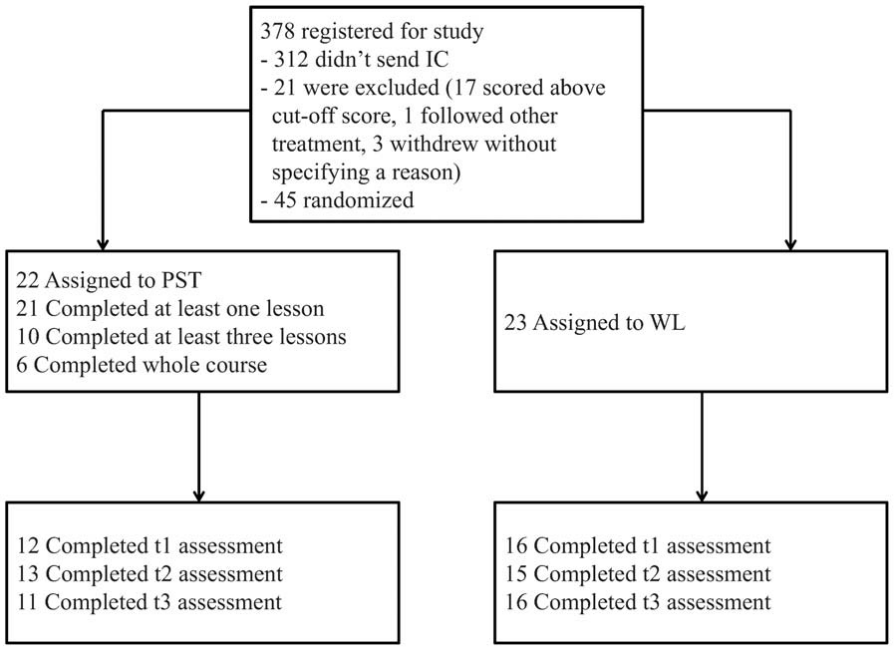
Progress of participants through the trial.

This study is a randomized controlled trial with two groups: an Internet-based guided self-help intervention (PST) and a waiting list control group (WL). The study was designed to compare the effects of the intervention with the waiting list. Based on a power of 0.80 and an alpha of 0.05, we needed 63 patients in each condition in order to be able to demonstrate moderate effects of *d* = 0.40. Therefore, the total sample size was set at 126. 45 participants were included in total (see **Fig. 1**). Participants on the waiting list got access to a website with general information on depression and anxiety, and could complete the intervention 4 months after baseline. Questionnaires were administered on the Internet and were sent after application (baseline) and 3 weeks, 5 weeks, and 4 months later.

### Randomization

Randomization took place at an individual level after the baseline measurement. We used block randomization, with each block containing 10, 12, 14, or 16 allocations. An independent researcher made the allocation schedule with a computerized random number generator. Immediately after randomization, adolescents were informed about the randomization outcome by email. If younger than 18 years of age, the subject's parents also received this email. We randomized 22 participants to the PST intervention and 23 participants to the waiting list condition.

### Measures

#### Structured diagnostic interview

Depression and anxiety subscales of the National Institute of Mental Health Diagnostic Interview Schedule for Children Version IV (NIMH DISC-IV) were conducted by telephone before randomization. The Diagnostic Interview Schedule for Children is a reliable and valid structured diagnostic interview designed for lay interviewers, which includes algorithms to diagnose DSM-IV disorders in children and adolescents [21]. Participants completed a telephone version of the generalized anxiety disorder, social phobia, panic, agoraphobia, major depression, and dysthymia modules of the NIMH DISC-IV. The NIMH DISC-IV assesses the presence of diagnoses occurring within both the past 12 months and the past 4 weeks. The interview was not a requisite of the study, 33 participants decided to participate. Telephone versions of structured psychiatric interviews in both adults [22] and youth [23] have been found to have a high correlation with in-person interviews.

#### Depressive symptoms

Depressive symptoms were assessed with the Centre for Epidemiologic Studies Depression scale (CES-D) [17]. The CES-D consists of 20 items for which subjects rate the frequency of symptoms during the past week with scores ranging from 0 (rarely or none of the time present [less than 1 day]) to 3 (most or all of the time present [5–7 days]), with a total score ranging between 0 and 60. A cut-off score of ≥16 can be used to identify adolescents with mild or greater levels of depression. The validity of the CES-D has been tested in different populations, including studies with adolescents [24]–[26]. In the current study, Cronbach's alpha ranged from 0.89 to 0.93.

#### Anxiety symptoms

To measure the prevalence of anxiety symptoms, the anxiety subscale of the Hospital Anxiety and Depression Scale (HADS-A) [18] was used. The HADS-A consists of seven items rated on a 4-point scale ranging from 0 (not at all) to 3 (a great deal of the time), with 3 indicating higher symptom frequency. A total score ranges from 0 to 21 and score ranges are classified as normal (0–7), mild (8–10), moderate (11–14) and severe (15–21). The HADS shows good homogeneity and reliability, with Cronbach's alpha ranging from 0.81 to 0.84 in different normal and clinical Dutch samples [27], and has been found valid and adequate for use with adolescents [28]. Cronbach's alpha in this study varied from 0.76 to 0.90.

#### Client satisfaction

The eight-item Client Satisfaction Questionnaire (CSQ-8) [29] assesses patient satisfaction. The CSQ-8 items can be scored on a scale from 1 (low satisfaction) to 4 (high satisfaction), with labels specific to particular items. The total CSQ score was obtained by averaging the items answered. Good construct validity and internal consistency have been previously reported [29]. Cronbach's alpha was 0.86 in this study.

In conformity with grade rating scales in Dutch education, participants were also asked to give an overall satisfaction rating on a scale from 1 (very bad) to 10 (excellent), followed by an open question on how this rating could have been improved.

#### Help-seeking behavior

To examine possible help-seeking in participants on the waiting list, adolescents were asked whether they searched for and received professional help (and from whom) for internalizing problems during the first 4 months of study participation.

### Statistical analyses

#### Missing values

All analyses were performed on the intention-to-treat sample. Baseline data were available for all participants. The Linear Mixed Modeling (LMM) procedure was used for all analyses to estimate missing values. LMM includes incomplete cases in the analysis and employs restricted maximum likelihood estimation to calculate parameter estimates. LMM assumes that missing data are missing at random.

#### Baseline differences and attrition

Baseline differences in demographic and clinical characteristics were investigated using Chi-square tests and t-tests. Attrition was defined as completing none or one of the three post measures.

### Effects

#### Treatment differences

LMM, using the variance components covariance structure, was used to investigate treatment differences. Time was treated as a continuous variable, condition was treated as a fixed effect, and the intercept was included as a random effect.

#### Recovery

For the CES-D total score, the standard cut-off score of 16 was used as an indication of recovery. A HADS score lower than 8 [30] was used as an indication of recovery. This was calculated only for those participants who did meet these criteria at baseline. The differences in recovery rate between the intervention and control group were also calculated with binary logistic regression.

#### Completion status

LMM was used to investigate differences in development of depressive symptoms between treatment completers, non-completers, and WL. Time was treated as a continuous variable. The intercept was included as a random effect. Completers were defined as respondents who completed all lessons. All analyses were conducted using SPSS for Windows version 17.0.

## Results

### Participants

Of the 378 adolescents who were potentially interested in participating, 312 adolescents did not give informed consent. From 66 adolescents we received baseline questionnaires and written informed consent. Of these, 17 did score above the cut-off on either the CES-D, HADS, or BDI suicide item, 1 already received different treatment, and 3 withdrew without specifying a reason. The remaining 45 participants were randomized. **Table 1** presents sample characteristics. Participants were mainly female (75.6%, n = 34). The mean age was 16.07 years (SD = 2.31). Almost all participants were of Dutch origin (91.1%, n = 41). The mean score of all participants on the CES-D at baseline was 25.02 (SD = 9.06, range = 5–40), on the HADS 8.84 (SD = 3.60, range = 2–14). Both averages are above cut-off scores for mild levels of depression and anxiety. In the 12 months before our baseline measurement, 45% of participants encountered any mood or anxiety disorder, with social phobia being the most common (42%). There were no statistically significant differences between the groups at baseline with respect to demographics, symptoms or diagnostic status (**Table 1** and **Table 2**).

**Table 1:** Sample characteristics at baseline.

	All(*n* = 45)	PST(*n* = 22)	WL(*n* = 23)	Statistic
**Age (years)**	16.07	15.78	16.36	t (43) = 0.84, *P* = 0.405
**Female**	34 (75.6%)	15 (68.2%)	19 (82.6%)	X^2^ = 1.27, *P* = 0.260
**Country of birth**				X^2^ = 0.002, *P* = 0.960
The Netherlands	41 (91.1%)	20 (90.9%)	21 (91.3%)	
**Education**				X^2^ = 5.43, *P* = 0.066
lower	17 (37.8%)	5 (22.7%)	12 (52.2%)	
middle	14 (31.1%)	7 (31.8%)	7 (30.4%)	
higher	14 (31.1%)	10 (45.5%)	4 (17.4%)	
**Diagnosis** *				
MD – past year	5 (15.2%)	3 (17.6%)	2 (12.5%)	X^2^ = 0.170, *P* = 0.680
MD – current	2 (6.1%)	0 (0%)	2 (12.5%)	X^2^ = 2.262, *P* = 0.133
GAD – past year	2 (6.1%)	0 (0%)	2 (12.5%)	X^2^ = 2.262, *P* = 0.133
GAD – current	1 (3.0%)	0 (0%)	1 (6.3%)	X^2^ = 1.096, *P* = 0.295
SP – past year	14 (42.4%)	9 (52.9%)	5 (31.3%)	X^2^ = 1.588, *P* = 0.208
SP – current	4 (12.1%)	1 (5.9%)	3 (18.8%)	X^2^ = 1.281, *P* = 0.258
PAN	3 (9.4%)	2 (12.5%)	1 (6.3%)	X^2^ = 0.368, *P* = 0.544

* Presence of diagnosis occurring within the past 12 months (“past year”) including the past 4 weeks (“current”). MD = Major Depression; GAD = Generalized Anxiety Disorder; SP = Social Phobia; PAN = Panic Disorder without Agoraphobia.

**Table 2:** Outcomes on depression and anxiety.

Measure and treatment condition	BaselineM (S.D.)	3 weeksM (S.D.)	5 weeksM (S.D.)	4 monthsM (S.D.)
**CES-D**				
PST	25.00 (8.22)	19.25 (7.21)	17.00 (9.17)	20.18 (10.39)
WL	25.04 (9.97)	21.88 (11.29)	17.47 (12.92)	19.75 (12.46)
**HADS**				
PST	8.63 (3.22)	7.17 (3.04)	6.84 (4.60)	6.64 (5.73)
WL	9.04 (3.99)	7.56 (4.70)	6.20 (5.72)	7.50 (5.11)

*Note*. M = Mean; S.D. = Standard Deviation; PST = Problem Solving Therapy group; WL = Waiting List control group.

### Attrition

Attrition rates for the full sample were 37.8% (n = 17) at the 3-week assessment, 37.8% (n = 17) at 5 weeks, and 40% (n = 18) at 4 months. Reasons for the high level of attrition were unknown. There were no significant differences in attrition rates between groups. Participants who completed post measures were younger than participants who didn't (15.46 and 16.89 years respectively, t(43) = 2.137, *P* = 0.038).

### Effects

#### Treatment differences

**Table 2** reports the means and standard deviations for depression and anxiety for the intervention and waiting list control group over 4 months. For depression, a significant overall improvement over time was found for the complete sample on the CES-D, Β = −2.31, SE = 0.86, *P* = 0.009. Neither significant group (Β = −0.10, SE = 3.58, *P* = 0.978) nor group x time interaction (Β = 0.54, SE = 1.14, *P* = 0.637) effects were found. Regarding anxiety, no significant time (Β = −0.73, SE = 0.39, *P* = 0.062), group (Β = −0.06, SE = 1.57, *P* = 0.968) or group x time interaction (Β = 0.25, SE = 0.51, *P* = 0.621) effects were found.

#### Recovery

36 participants suffered clinical levels of depression according to the CES-D at baseline (80% of total sample). 37.5% of participants who completed post-measures had recovered at 5 weeks and 25% had recovered at 4 months across both groups. Regarding anxiety, 31 participants suffered clinical levels at baseline (68.9%). After 5 weeks, 60% had recovered while 52.4% had recovered after 4 months. Recovery effects were not significantly different between intervention and waiting list groups.

#### Treatment completers versus non-completers

Many participants failed to complete the whole course. Of those participants assigned to the intervention condition, 10 (45%) participants completed three or more lessons and 6 (27%) completed all five. All but 1 participant completed the first lesson. Completers were younger than non-completers (14.83 years versus 16.94 years), t (20) = −2.20, *P* = 0.04. Regarding symptom measures at baseline, no differences were found between completers and non-completers.

We investigated differences in development of depression and anxiety scores between treatment completers, non-completers, and waiting list condition. No differences were found in improvement of depressive and anxiety symptoms between completers and non-completers. Moreover, we examined differences in development of depression and anxiety between participants who completed three or more lessons, participants who completed less than three lessons, and participants on the waiting list. No differences were found in improvement of depression and anxiety.

#### Client satisfaction

**Table 3** provides an overview of the CSQ items and the total scale score that were used to estimate participants' satisfaction with the intervention. Given the 1–4 metric for the CSQ scale, participants expressed moderate satisfaction (total score M = 2.51, SD = 0.55). The mean overall satisfaction grade on a 1–10 scale was 6.45 (SD = 1.28) and 85% of participants reported grades of 6 or above. Fourteen participants provided suggestions on how to improve the intervention which have been abstracted into five main suggestions. Suggestions were: better communication and guidance, more feedback from supervisor and receiving emails when having received feedback (adolescents now had to be logged onto the website in order to see whether they got a response on their assignments) (n = 5), more clarity on what to do and why, a clearer website (n = 3), more exercises and more elaborative on exercises, more exercises in negative thinking (n = 3), more time to work on exercises, extension of course length (n = 2), more originality in self-help program (n = 1).

**Table 3:** Means and standard deviations (S.D.) for each CSQ item and total scale score (n = 21).

Item or scale	Mean	S.D.
Rate quality of service	2.67	0.66
Got the kind of service wanted	2.29	0.90
Program met needs	2.43	0.75
Recommend to a friend	2.23	0.73
Satisfaction with help received from supervisor	2.29	0.90
Helped deal more effectively with problems	2.76	0.77
How satisfied with service	2.86	0.73
Would come back to the program	2.33	0.73
Total scale score	2.51	0.55

#### Help-seeking

Eleven adolescents on the waiting list responded on the question about possible help-seeking behavior during study participation. Two adolescents had received professional help from a psychologist.

## Discussion

This is the first RCT examining the effects of preventive Internet-based problem solving therapy (PST) for adolescents with depressive and anxiety symptoms. Internet-based PST was compared to a waiting list control group and intervention effects were investigated over 4 months. Results show that depressive and anxiety symptoms declined in both groups. However, no support was found for the effects of Internet-based PST in reducing depression and anxiety in comparison to the waiting list control group, but the small sample size and high drop-out rate precluded meaningful interpretation of this finding.

Previous research into the problem-solving treatment with an adult population has found that the program obtains significant effects [13], [31]. Several explanations may be considered for not finding any additional effect of PST above usual care for our adolescent population. One reason for the lack of effects in this study is that our intervention was not implemented precisely in the way it was intended due to website problems. That is, the website content was not presented in an optimally format which could have caused difficulties for adolescents in understanding and working through the intervention. Moreover, the intervention may have lacked sufficient guidance and email support for some participants because of technical difficulties in the email support module. Six participants have encountered delayed feedback. Both suppositions are supported by satisfaction reports and may have impacted completion status. The level of completers in our study (27%) was relatively low compared to adult studies into web-based self-help [31]–[33]. Differences in depression and anxiety outcomes between completers and non-completers were not found, however.

Another reason for the non-significant findings here may be reduced appropriateness of our intervention for this age group. The self-help and individual format of our intervention requires extensive self-discipline and motivation, and this may be too informal in nature for treatment of adolescents. Also, depression impacts on individuals' motivation and their ability to concentrate in a self-directed format [10]. It is also possible that PST may not be attractive enough for adolescents, or the intervention may have been of too high difficulty or of insufficient duration for problem solving skills to become internalized to produce lasting effects.

A different possible reason for the lack of effects may be participants' severity of symptom levels at baseline. To illustrate, most adolescents reported clinically significant levels of depression or anxiety and several had encountered a mood or anxiety disorder in the past 12 months. Possibly, the PST intervention is not suitable for adolescents with more severe symptoms.

Finally, we should note that participants in the waiting list control condition may have attended other treatments during the study, possibly affecting our results. Two out of 11 waiting-list participants received other treatment. Although it is not clear to what extent this may have affected the results, our results are vulnerable to many influences considering our small sample size.

The present study has a number of significant strengths. The study is one of only a small number of randomized controlled trials to date into web-based interventions for adolescents with depression and anxiety. The intervention being examined required minimal training of therapists and was relatively cheap to administer, thereby increasing the probability that the intervention would be sustained and distributed within mental health care services. Moreover, reliable and valid measures of depressive and anxiety symptoms were used, recruitment was widespread, and diagnostic status at baseline was assessed. However, limitations should be noted. First, the sample size was very small and questions could be raised about the representativeness of the sample and the generalizability of our findings to the wider population. Specifically, immigrants were fairly underrepresented among participants, and we predominantly excluded early and middle adolescents who were reluctant to request parental consent. It is likely that participants differed in other important ways from those who did not sign up for the study. Second, in common with many other online interventions [34], we were faced with a high attrition rate, possibly due to adolescents losing interest in participating in the study. Another possible limitation is the use of a wait-list control group in that it is unclear whether the prospect of receiving an intervention within a couple of months could have influenced results. A final limitation is that demand characteristics, such as receiving a telephone call when reporting possible severe depressive or anxiety symptoms, may have affected participant responses to the questionnaires.

Overall, the results of this study do not provide support for the use and implementation of an Internet-based problem-solving therapy for adolescents with depressive and anxiety symptoms. Several explanations for this lack of effect may be provided. In general, this study may have had inadequate power to demonstrate effects and further research is needed to establish whether web-based PST will be effective in adolescents if adapted or aimed at a specific subgroup of adolescents. More research is required to ascertain what intervention content, amount, timing, and duration of delivery is most effective and for which group of adolescents [35], as well as the potential of Internet-based interventions for adolescents with internalizing symptoms.

## Supporting Information

Checklist S1 **CONSORT Checklist.**
(DOC)

Protocol S1 **Trial Protocol.**
(PDF)
